# Sickle cell disease and malaria: decreased exposure and asplenia can modulate the risk from *Plasmodium falciparum*

**DOI:** 10.1186/s12936-020-03212-w

**Published:** 2020-04-25

**Authors:** Richard O. Mwaiswelo, William Mawala, Per O. Iversen, Mariane de Montalembert, Lucio Luzzatto, Julie Makani

**Affiliations:** 1 Department of Microbiology, Immunology and Parasitology, Hubert Kairuki Memorial University, Dar es Salaam, Tanzania; 2 Department of Parasitology and Medical Entomology, Muhimbili University of Health and Allied Sciences, Dar es Salaam, Tanzania; 3 Department of Hematology and Blood Transfusion, Muhimbili University of Health and Allied Sciences, Dar es Salaam, Tanzania; 4 Department of Haematology, Oslo University Hospital, Oslo, Norway; 5 Department of General Pediatrics and Pediatric Infectious Diseases, Necker-Enfants malades Hospital, Assistance Publique-Hôpitaux de Paris, Université de Paris, Paris, France

**Keywords:** Chemoprophylaxis, Malaria, Sickle cell disease, Splenic dysfunction, Splenectomy

## Abstract

**Background:**

Patients with sickle cell disease (SCD), an inherited haemoglobinopathy, have increased risk of malaria, at least in part due to impaired splenic function. Infection with *Plasmodium falciparum* in SCD patients can trigger painful vaso-occlusive crisis, increase the severity of anaemia, and contribute to early childhood mortality.

**Case presentation:**

A 17 year-old Tanzanian male with known SCD was admitted to Muhimbili National Hospital, a tertiary referral centre in Dar-es-Salaam, following an attack of malaria. From 2004 to 2007 the patient had lived in USA, and from 2010 to 2016 in France where, on account of hypersplenism and episodes of splenic sequestrations, in 2014 the spleen was removed. After appropriate clinical and laboratory assessment the patient was re-started on hydroxyurea; and anti-malarial-prophylaxis with proguanil was instituted. The patient has remained well and malaria-free for the following 15 months.

**Conclusion:**

SCD patients are highly vulnerable to malaria infection, and impaired splenic function is a feature of SCD patients, even in those who still anatomically have a spleen. This patient had a surgical splenectomy and, in addition, had probably lost some of the acquired malaria-immunity by having lived for several years in malaria-free areas. This patient is a compelling reminder that long-term anti-malarial prophylaxis should be offered to all patients with SCD who live in malaria-endemic areas.

## Background

Sickle cell disease (SCD) is an autosomal recessive disorder characterized by chronic haemolytic anaemia and painful vaso-occlusive crises. SCD is common in sub-Saharan Africa, with a birth prevalence that in some areas reaches 2% [1], and where it is therefore a true issue of public health importance. Without early diagnosis and appropriate care many children born with SCD do not survive beyond their fifth birthday [1, 2].

SCD patients also experience increased susceptibility to severe infections, including malaria [3, 4]. This is particularly important because the geographic distribution of SCD parallels closely that of malaria, both disorders being frequent in the entire tropical belt of Africa, including Tanzania [5, 6]. This parallel epidemiology is not a coincidence: rather, there is evidence that heterozygotes for the sickle gene (Hb genotype AS) are relatively protected against death due to malaria, probably through accelerated clearance by macrophages of *Plasmodium falciparum*-infected erythrocytes [7]. On the other hand, homozygous (Hb SS) SCD patients are at increased risk of dying from malaria [3, 4, 7].

Splenic dysfunction may be responsible for increased susceptibility of SCD patients to malaria as well as to other infections [8, 9], as removal of abnormal erythrocytes and of intra-erythrocytic inclusions, including parasites, is one of the main functions of the spleen [10, 11]. Therefore, persons who are either splenectomized or who have impaired splenic function have increased susceptibility to malaria [11, 12]. Immunity against malaria is complex, but in malaria-endemic areas some degree of immunity develops regularly. This may be weakened when recurrent exposure ceases, for example in a person who has moved to a malaria-free area [13].

In view of the higher malaria mortality rate in SCD patients, although it is questionable whether anti-malarial prophylaxis reduces the frequency of vaso-occlusive crises [14], this prophylaxis is recommended [15], but it is far from universally practiced in sub-Saharan countries [1, 5]. This is true in Tanzania where anti-malarial chemoprophylaxis for SCD patient is an established policy that, however, is far from being regularly implemented.

This is a report of a patient with SCD who was asplenic, who had lived for several years in malaria-free environment and who, upon return to Tanzania, did not initially receive anti-malarial prophylaxis and suffered recurrently from malaria.

### Case presentation

A 17 year-old Tanzanian male with known SCD came in May 2018 to the Sickle Cell Clinic at the Muhimbili National Hospital (MNH) in Dar-es-Salaam, Tanzania with a complaint of loss of appetite and nausea. The day before the patient had been discharged from a local hospital in Dar-es-Salaam where, having been admitted 5 days previously because of fever, nausea, loss of appetite, generalized body pain and weakness. *Plasmodium falciparum* malaria infection was diagnosed upon microscopic examination of blood slides. The patient was treated with artesunate injection followed by quinine tablets. A similar episode, with confirmed *P. falciparum* infection, had occurred some weeks earlier, when the patient had been treated with artemether-lumefantrine tablets.

The medical history showed that the patient had been diagnosed with SCD at the age of 2 years. At the age of 5 years when living in the USA, the patient was started on hydroxyurea. In 2007, he returned to Tanzania where, in 2008, he had an episode of malaria. In 2010, the patient moved to France where further investigations revealed that his SCD was sickle beta thalassaemia, and that he was also homozygous for alpha-thalassaemia. The beta thalassaemia mutation was identified as IVS1nt5G > C. Deficiencies of coagulation factors VII and X were also detected. His steady-state Hb level was 10–11 g/dL; however, he required blood transfusion on 9 occasions because of recurrent exacerbations of anaemia, attributed to hypersplenism and recurrent splenic sequestration. Therefore in 2014 he had a laparoscopic splenectomy. The spleen weighed 428 g and histology showed marked congestion without haemosiderosis or other abnormalities.

In 2016, the patient returned to Tanzania where he now attends secondary school. In 2017 the patient was feeling so well that he stopped taking hydroxyurea, his steady-state haemoglobin being 9–10 g/dL, and his only medication was folic acid (5 mg/day). However, recently he has had several bone pain crises.

On admission to MNH in May 2018 clinical examination did not reveal any specific findings. There was no severe pain and no evidence of infection. The patient’s Hb was 7.3 g/dL and a malaria test was negative. The patient continued on folic acid, hydroxyurea (500 mg/day) was re-introduced, and anti-malarial prophylaxis with proguanil (100 mg/day) was started. On monthly follow-up visits from June 2018 to September 2019 the patient has been generally well, except for occasional pain that responded to paracetamol, diclofenac or ibuprophen. Laboratory findings (Table 1) were stable.

**Table 1:** Laboratory findings obtained at hospital admission and outpatient follow-up visits

Laboratory values	Admission MNH (may 2018)	Visit 1 (june 2018)	Visit 2 (july 2018)	Visit 3 (aug 2018)	Visit 4 (sept 2018)	Visit 5 (march 2019)	Visit 6 (sept 2019)
Haemoglobin (g/dL)	7.3	8.5	10.8	10.4	10.5	10.8	11.2
Neutrophils (× 10^9^/L)	2.8			6.1			4.3
Lymphocytes (× 10^9^/L)	0.8			2.6			2.4
Monocytes (× 10^9^/L)	0.3			0.5			0.7
Eosinophils (× 10^9^/L)	0.03			0.1			0.1
Platelets (× 10^9^/L)	384			344			265
Beta-glutamyl transferase (U/L)				25.5			
Lactate dehydrogenase (U/L)				343			
Alanine aminotransferase (U/L)				19	20		
Aspartate aminotransaminase (U/L)				26			
Bilirubin-total (mg/dL)				1.13	1.31		
Creatinine (mg/dL)				0.73	0.81		

*MNH* Muhimbili National Hospital, *Hb* haemoglobin

A high performance liquid chromatography haemoglobin analysis confirmed in this patient the diagnosis of sickle beta thalassaemia (Table 2). The mother is an A/S heterozygote and the father is a beta-thalassaemia heterozygote. Surprisingly, the patient’s sister, who reports having been generally well, was also found to have sickle beta thalassaemia.

**Table 2:** Blood profiles of patient and his family members

Laboratory values	Patient	Mother	Father	Sister
Blood values				
Hb (g/dL)	10.4	10.0	11.0	9.4
MCV (fL)	76.0	66.5	67.9	61.0
MCHC (g/dL)	34.7	31.6	31.9	33.6
Reticulocytes	97.1	NA	72.6	NA
RDW (%)	40.0	42.2	36.7	39.9
Neutrophils (× 10^9^/L)	3.7	3.0	4.6	4.8
Platelets (× 10^9^/L)	294	342	221	126
Blood film	Aniso-, poikilo-, elliptocytosis, very rare ISC hypochromic cells.	Anisocytosis, target cells,	Anisocytosis, microcytosis	Aniso-, poikilo-, elliptocytosis, target cells
HPLC-data of Hb-subtypes (%)				
A	6.3	57.7	79.5	5.3
A_2_	6.8	2.7	4.1	5.6
F	12.0	3.3	12.0	10.2
S	73.0	34.5	ND	79.0
Other	ND	ND	4	ND
Interpretation	Sickle beta-thalassaemia double heterozygote	AS heterozygote	Beta -thalassaemia heterozygote	Sickle beta-thalassaemia double heterozygote

Values were measured in October 2019

*Hb* haemoglobin, *MCV* mean cell volume, *MCHC* mean cell Hb concentration, *RDW* red cell distribution width, *HPLC* high pressure liquid chromatography, *NA* not available, *ND* not determined

## Discussion

Malaria infection may be fatal in any person: however, the risk of fatality is influenced by several genetic and acquired factors. Among the former, SCD is one of the foremost: it increases greatly the mortality from malaria infection; probably in large part because the *P. falciparum*-induced destruction of both parasitized and non-parasitized erythrocytes will precipitate a severe exacerbation of the already existing chronic anaemia [16, 17]. This is in striking contrast to the well known significant protection enjoyed by haemoglobin S heterozygotes (sickle cell trait) against *P. falciparum* malaria [7], and the triumph of good over evil: protection by the sickle gene against malaria [18]. In SCD malaria can also trigger painful vaso-occlusive crisis in SCD patients and it is among the leading causes of hospital admissions and mortality in endemic countries [3, 13].

As for acquired factors, partial immunity against malaria is usually acquired during the first 5 years of life, is dependent on the intensity of transmission and, therefore, on exposure frequency [19], and it tends to wane off with time if re-exposure does not occur [19, 20]. A previous study has found that immigrants of African origin who had lived in Sweden (a malaria-free country) for 15 years or more, once they returned to Africa had a risk of malaria infection and of severe malaria similar to that of non-African travellers [20]. In the defense against malaria a significant role is played by the spleen because, by virtue of its unique anatomy, it can retain parasitized erythrocytes (as well as non-parasitized, but otherwise abnormal erythrocytes); indeed, impaired splenic function reduces the clearance rate of malaria parasites from the circulation [8, 10, 11]. In SCD by early adulthood auto-splenectomy or functional asplenia is the rule rather than the exception: but, compared to homozygous sickle cell anaemia (SS), in sickle beta-thalassaemia the spleen is much more likely to persist and to remain enlarged even in adults. This may be associated with pooling of blood in the spleen that intermittently becomes greater (splenic sequestration) [21], whereby indications for splenectomy are more often compelling in sickle beta-thalassaemia compared to homozygous sickle cell anaemia.

In this patient each one of these factors seems to have conspired with the others: (i) The patient had sickle beta thalassemia, a well known form of SCD; (ii) he was asplenic; and (iii) after being born and having lived in Tanzania for the first 4 years of his life, he lived for a total of 8 years in malaria-free countries (USA and France), whereby whatever immunity he had previously developed may have faded. He had at least two documented episodes of malaria infection prior to his splenectomy, and at least two after splenectomy. In this patient, therefore, the spleen was removed for good clinical reasons, and the procedure was recommended only after a balanced judgment of pros and cons. One goal was to reduce substantially the rate of blood transfusion, and this was achieved. As for the implications of being asplenic, it must be accepted that living in a malaria-endemic region is an added risk.

Malaria episodes in this patient have been treated promptly: fortunately, because in a splenectomized SCD patient it might rapidly become life-threatening, a decision was made to institute anti-malarial chemoprophylaxis [15]. Officially recommended regimens in Tanzania include weekly mefloquine, daily atovaquone/proguanil, daily doxycycline, or weekly artemisinin-based combination therapy. None of these options is without side effects [22], in addition, the patient could not afford any of them. Hence, a widely used proguanil monotherapy drug was prescribed [23, 24]. Since Dar-es-Salaam is regarded as a low malaria transmission area, prophylaxis cannot be automatically credited with having prevented further episodes of malaria infection, but in the 18 months since starting proguanil the patient has had no malaria recurrence, no fever and no hospital admission. Since his last hospitalization in May 2018, his Hb level has risen significantly, which may be attributed to the combined effects of not having malaria infection and having resumed hydroxyurea therapy. Nonetheless, this case report illustrates what can happen (and probably often does happen) when anti-malarial chemoprophylaxis is not provided.

## Conclusion

This patient was a living reminder that if SCD cannot be cured, then at least patients with SCD should be protected from *falciparum* malaria, an exogenous extra threat that is preventable.

## Data Availability

The data collected during the current study are not publicly available due the privacy of the patient, but are available from the corresponding author on reasonable request.

## Abbreviations

SCD: Sickle cell anaemia
Hb: Haemoglobin
HbSS: Homozygote for the sickle cell gene
HbAS: Heterozygote for the sickle cell gene
MNH: Muhimbili National Hospital
